# Absence of single nucleotide polymorphisms (SNPs) in the open reading frame (ORF) of the prion protein gene (*PRNP*) in a large sampling of various chicken breeds

**DOI:** 10.1186/s12864-019-6315-8

**Published:** 2019-12-03

**Authors:** Yong-Chan Kim, Sae-Young Won, Byung-Hoon Jeong

**Affiliations:** 1 Korea Zoonosis Research Institute, Chonbuk National University, 820-120 Hana-ro, Iksan, Jeonbuk 54531 Republic of Korea; 2 Department of Bioactive Material Sciences, Chonbuk National University, Jeonju, Jeonbuk 54896 Republic of Korea

**Keywords:** Chicken, Prion protein gene, *PRNP*, Hexapeptide repeat, Prion

## Abstract

**Background:**

Prion diseases are zoonotic diseases with a broad infection spectrum among mammalian hosts and are caused by the misfolded prion protein (PrP^Sc^) derived from the normal prion protein (PrP^C^), which encodes the prion protein gene (*PRNP*). Currently, although several prion disease-resistant animals have been reported, a high dose of prion agent inoculation triggers prion disease infection in these disease-resistant animals. However, in chickens, natural prion disease-infected cases have not been reported, and experimental challenges with prion agents have failed to cause infection. Unlike other prion disease-resistant animals, chickens have shown perfect resistance to prion disease thus far. Thus, investigation of the chicken *PRNP* gene could improve for understanding the mechanism of perfect prion-disease resistance. Here, we investigated the genetic characteristics of the open reading frame (ORF) of the chicken *PRNP* gene in a large sampling of various chicken breeds.

**Results:**

We found only tandem repeat deletion polymorphisms of the chicken *PRNP* ORF in the 4 chicken breeds including 106 Dekalb White, 100 Ross, 98 Ogolgye and 100 Korean native chickens. In addition, the distribution of chicken insertion/deletion polymorphisms was significantly different among the 4 chicken breeds. Finally, we found significant differences in the number of *PRNP* SNPs between prion disease-susceptible species and prion disease-resistant species. Notably, chickens lack SNPs in the ORF of the prion protein.

**Conclusion:**

In this study, we found that the absence of SNPs in the chicken *PRNP* ORF is a notable feature of animals with perfect resistant to prion disease.

## Background

Prion diseases are zoonotic diseases caused by the misfolded prion protein (PrP^Sc^) derived from the normal prion protein (PrP^C^) and have a broad infection range in mammalian hosts, including ruminants and humans [1–9]. To date, prion disease-affected cases have not been reported in various bird species; however, normal functions of PrP^C^ and tandem repeat domains (octapeptide in mammals and hexapeptide in birds) are well known and conserved in both mammals and birds, respectively [10, 11]. The function of PrP^C^ may be related to regulation of stress protection, myelin maintenance, circadian rhythm, mitochondrial homeostasis and metal-ion homeostasis [12]. Among these functions, metal-ion homeostasis was strictly linked to the tandem repeat domain of prion protein [10].

In mammals, numerous single nucleotide polymorphisms (SNPs) in the open reading frame (ORF) of the prion protein gene (*PRNP*) have been identified thus far. Among these SNPs, several prion disease-associated SNPs have been reported in prion disease-susceptible species, including humans, sheep and goats. In particular, the *PRNP* codons 129 and 219 in humans are strongly associated with CJD susceptibility [13, 14]. In addition, the *PRNP* codons 136, 154 and 171 in sheep [15, 16] and the *PRNP* codons 127, 142, 143, 146, 154, 171, 211 and 222 in goats are also correlated with the incidence of scrapie [17–21]. Approximately 40 SNPs of *PRNP* ORF have been reported in cattle. However, prion disease-related polymorphisms have not been reported thus far [22–25]. In addition, dog, which is known as prion disease resistant animal, showed very little polymorphisms in the *PRNP* ORF [26]. Although real challenge study has not been performed to confirm the transmission of prion disease to horse, horse prion protein transgenic mouse showed the resistance to infection of several agents of prion diseases, including RML, scrapie, chronic wasting disease (CWD), transmissible mink encephalopathy (TME) and bovine spongiform encephalopathy (BSE) [27]. In addition, horse prion protein had high structural stability and its horse specific amino acids showed the protective effect against prion disease [28, 29]. These results suggest that horse is a prion disease-resistant animal. Interestingly, horse has only one SNP in the ORF of the *PRNP* gene [5].

Our previous study indicated that genetic polymorphisms, which are considered genetic susceptibility factors in mammals, were significantly different in the chicken prion *PRNP* gene. Chickens lack SNPs and have only one insertion/deletion polymorphism located on the hexapeptide repeat domain in the prion protein. However, in a study performed with only one chicken breed, Dekalb White, it was unclear whether this is a genetic characteristic of chickens or a characteristic of a chicken breed, Dekalb White [30].

Here, we performed direct sequencing of the chicken *PRNP* gene and confirmed the genetic polymorphism of the chicken *PRNP* gene in 4 chicken breeds, including Dekalb White, Ross, Ogolgye and Korean native chickens. We also compared the genotype and allele distribution of the chicken *PRNP* polymorphisms among 4 chicken breeds. Lastly, we investigated and compared SNPs of prion disease-susceptible species (humans, sheep, goats and cattle) and prion disease-resistant species (horses and chickens).

## Results

To identify genetic polymorphisms of the chicken *PRNP* gene, we performed direct sequencing in 298 chickens including 3 chicken breeds (100 Ross, 100 Korean native chickens and 98 Ogolgye). Interestingly, we found only the c.163_180delAACCCAGGGTACCCCCAT (p.55_60delNPGYPH) polymorphism in the 3 chicken breeds (Ross, Ogolgye, and Korean native chickens). The c.268_269insC polymorphism, which was found in the Dekalb White breed, was not found in the 3 studied chicken breeds (Tables 1 and 2 and Additional file 1).

**Table 1:** Comparison of genotype and allele distributions of chicken *PRNP* c.163_180delAACCCAGGGTACCCCCAT (p.55_60delNPGYPH) polymorphism in 4 chicken breeds

Breeds	Total, n	Genotype frequency, n (%)			*P*-value	Allele frequency, n (%)		*P*-value	HWE	Ref
		WT/WT	WT/DEL	DEL/DEL		WT	DEL			
Dekalb White	106	13 (12.3)	89 (84)	4 (3.7)	–	115 (54.2)	97 (45.8)	–	<0.001	[22]
Ross	100	84 (84)	16 (16)	0 (0)	<0.0001	184 (92)	16 (8)	<0.0001	0.3845	This study
Ogolgye	100	68 (68)	31 (31)	1 (1)	<0.0001	167 (83.5)	33 (16.5)	<0.0001	0.2112	This study
Korean native chickens	100	82 (82)	17 (17)	1 (1)	<0.0001	181 (90.5)	19 (9.5)	<0.0001	0.9097	This study

**Table 2:** Genotype and allele frequencies of the chicken *PRNP* c.268_269insC polymorphism in 4 chicken breeds

Breeds	Total, n	Genotype frequency, n (%)			*P*-value	Allele frequency, n (%)		*P*-value	HWE	Ref
		WT/WT	WT/INS	INS/INS		WT	INS			
Dekalb White	106	102 (96.2)	4 (3.8)	0 (0)	–	208 (98.1)	4 (1.9)	–	0.8431	[22]
Ross	100	100 (100)	0 (0)	0 (0)	0.122	200 (100)	0 (0)	0.1238	NA	This study
Ogolgye	100	100 (100)	0 (0)	0 (0)	0.122	200 (100)	0 (0)	0.1238	NA	This study
Korean native chickens	100	100 (100)	0 (0)	0 (0)	0.122	200 (100)	0 (0)	0.1238	NA	This study

*NA* Not Available

Next, we compared the genotype and allele frequencies of two chicken *PRNP* polymorphisms in 4 chicken breeds, Dekalb White, Ross, Ogolgye and Korean native chickens. Detailed values of genotype and allele frequencies of the c.163_180delAACCCAGGGTACCCCCAT polymorphism of the chicken *PRNP* gene are described in Table 1. Except for Dekalb White, all chicken breeds were in HWE. Interestingly, significant differences in genotype and allele distributions were found among Dekalb White and Ross (*p* < 0.001), Ogolgye (*p* < 0.001) and Korean native chickens (*p* < 0.001) (Table 1). In addition, we compared the genotype and allele distributions of the c.268_269insC polymorphism among 4 chicken breeds. Notably, the WT/INS genotype and insertion allele were only identified in 4 out of 106 Dekalb White chickens (3.8%). There were similar distributions in genotype (*p* = 0.122) and allele (*p* = 0.1238) frequencies of the c.268_269insC polymorphism between the Dekalb White and 3 chicken breeds (Table 2).

Furthermore, we analyzed haplotypes of the two insertion/deletion polymorphisms among the 4 chicken breeds. Detailed degrees of haplotype distribution in the 4 chicken breeds are described in Table 3. Three major haplotypes were found, and the ht3 haplotype was only detected in the Dekalb White breed. Statistically different distributions of the haplotypes were found between Dekalb White and Ross (*p* < 0.0001), Ogolgye (p < 0.0001) and Korean native chickens (*p* < 0.0001).

**Table 3:** Haplotype frequencies of two *PRNP* polymorphisms in 4 chicken breeds

Haplotypes	c.163_180del AACCCAGGGTACCCCCAT	c.268_269ins C	Dekalb White	Ross	Ogolgye	Korean native chickens
ht1	WT	WT	115 (54.2)	184 (92)	167 (83.5)	181 (90.5)
ht2	DEL	WT	93 (43.9)	16 (8)	33 (16.5)	19 (9.5)
ht3	DEL	INS	4 (1.9)	0 (0)	0 (0)	0 (0)
P-value			–	<0.0001	<0.0001	<0.0001
Ref			[21]	This study	This study	This study

**Fig. 1:**
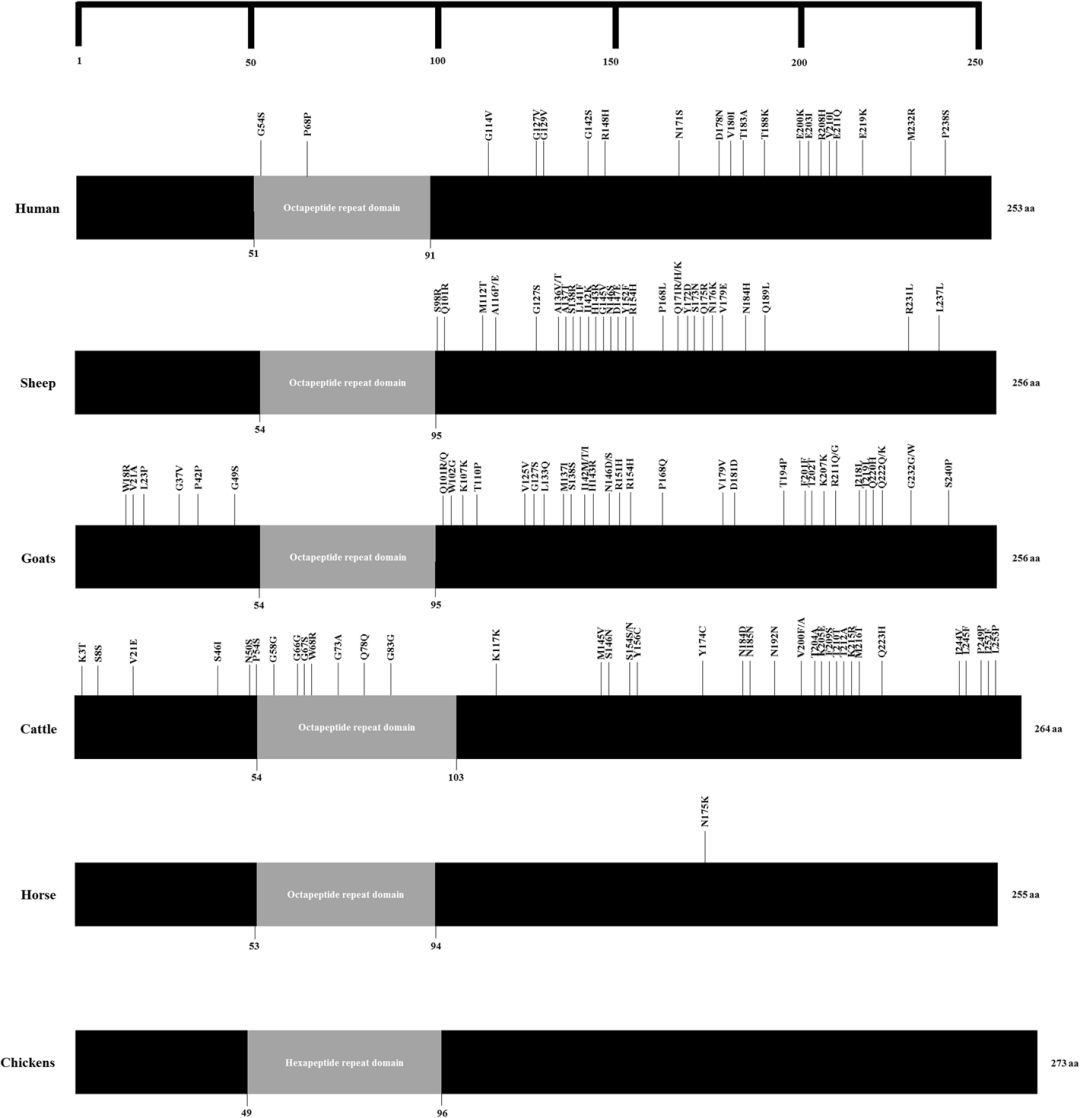
Distribution of the single nucleotide polymorphisms (SNPs) at coding region of the prion protein gene (*PRNP*) in various species. Previously reported SNPs at coding region of *PRNP* gene in human, sheep, goats, cattle, horse and chickens. Edged horizontal bar indicates the length of amino acids. Gray boxes indicate tandem repeat domains

Lastly, we surveyed SNPs of the *PRNP* gene in prion disease-susceptible species (humans, sheep, goats, and cattle) and prion disease-resistant species (horses and chickens). Over 20 SNPs were found in prion disease-susceptible species. However, in prion disease-resistant animals, only one SNP (N175K) and zero SNPs have been reported in horses and chickens, respectively (Table 4, Fig. 1).

**Table 4:** Distribution of the single nucleotide polymorphisms (SNPs) at the open reading frame (ORF) of the prion protein gene (*PRNP*) in various species

Species	Polymorphisms	Total, n	Ref.
Human	G54S, P68P, G114 V, G127 V, M129 V, G142S, R148H, N171S, D178N, V180I, T183A, T188K, E200K, V203I, R208H, V210I, E211Q, E219K, M232R, P238S	20	[6, 31, 32]
Sheep	S98R, Q101R, M112 T, A116P, A116E, G127S, A136V, A136T, M137 T, S138R, L141F, I142K, H143R, G145 V, N146S, D147E, Y152F, R154H, P168L, Q171R, Q171H, Q171K, Y172D S173 N, Q175R, N176K, V179E, N184H, Q189L, R231R, L237 L	31	[33–36]
Goats	W18R, V21A, L23P, G37 V, P42P, G49S, Q101R, Q101Q, W102G, K107K, T110P, V125 V, G127S, L133Q, M137I, S138S, I142M, I142T, I42I, H143R, N146D, N146S, R151H, R154H, P168Q, V179 V, D181D, T194P, F201F, T202 T, K207K, R211Q, R211G, I218L, T219 L, Q220H, Q222K, Q222Q, G232 W, G232G, S240P	41	[37, 38]
Cattle	K3T, S8S, V21E, S46I, N50S, P54S, G58G, G66G, G67S, W68R, G73A, Q78Q, G83G, K117K, M145 V, S146 N, S154S, S154 N, Y156C, Y174C, N184D, N185 N, N192 N, V200F, V200A, T204A, K205E, F209S, T210 T, T212A, K215R, M216 T, Q223H, I244V, L245F, P249P, I252F, L253P	38	[22–25]
Horse	N175K	1	[5]
Chickens	ND^a^	0	[30], This study

^a^*ND* Not Detected

## Discussion

In the present study, we confirmed that the chicken *PRNP* gene has only an insertion/deletion polymorphism at the tandem repeat domain in 4 chicken breeds. Because numerous case-control studies have found that SNPs of the *PRNP* gene play a pivotal role in prion pathogenesis, the absence of SNPs in the chicken *PRNP* gene is noteworthy. In addition, we identified significant differences in the genetic distribution of tandem repeat polymorphisms among Korean indigenous chickens (Korean native chicken and Ogolgye) and nonindigenous chickens (Dekalb White and Ross). According to previous studies, the tandem repeat domain is a direct binding site of metal ions, especially copper [10, 39–41]. Because the tandem repeat domain is a functional domain of the prion protein, further analysis of the highly polymorphic state of the chicken *PRNP* gene is needed in the future. Indeed, in a recent study, prion protein was expressed in lung epithelial cells and protected apoptosis induced by influenza A viruses. The lung in the prion protein knockout mouse model showed severe damage, higher levels of reactive oxygen species and cleaved caspase-3. In addition, animals with transgene for prion protein to a tandem repeat deleted model were also highly susceptible to influenza A virus infection. The results indicate that prion protein is also important in the susceptibility of influenza A viruses [42]. A previous study reported that chicken prion mRNA was overexpressed in Marek’s disease-infected chicken embryo fibroblasts and that knockdown of chicken prion protein in Marek’s disease virus-infected avian T cells reduced cell viability. This finding implied that Marek’s disease-induced tumors can be inhibited by reduced expression levels of the prion protein [43]. In a recent study, because the number of tandem repeat domains has been directly connected with the functional capacity of the prion protein, our findings in this study can also be related to the malignancy of Marek’s disease-induced tumor [43]. Thus, according to the genotype distribution of the functional domain of the chicken *PRNP* gene found in this study, investigation of the susceptibility to influenza A and the malignancy of Marek’s viruses is highly desirable in the future.

In addition, we confirmed the absence of SNPs in the ORF of the chicken *PRNP* gene in a large sampling of various chicken breeds. Notably, although the origins of the evaluated chickens are geographically divided (Korean native chicken: Korea; Ogolgye: China; Ross: Scotland; and Dekalb White: U.S.A), all chicken breeds showed common genetic characteristics of the *PRNP* gene. Since chicken showed high rate of SNPs in other genes except for *PRNP* gene, the absence of SNPs of the chicken *PRNP* gene is remarkable [44, 45]. In addition, natural prion disease-infected cases have not been reported in chickens, and parenteral and oral challenge of prion agent showed the failure of infection [46]. In other words, unlike other prion disease-resistant animals, including horses and dogs, chickens demonstrate perfect resistance to prion disease thus far [26]. Thus, the genetic characteristic of the absence of SNPs in the chicken *PRNP* gene is a notable features of prion disease-resistant animals. A good approach to understanding the pathogenesis of prion disease may be through the analysis of chicken-specific amino acids in interspecies-conserved domains of prion proteins. Although insertion/deletion polymorphism in the octapeptide repeat domain of bovine prion protein has been reported in cattle, this polymorphism has never been associated with the susceptibility or resistance of prion disease [22–25, 47–49]. However, the variations of octapeptide repeat domain of human prion protein confer the susceptibility to prion disease in human [6, 31]. Amino acid sequences in hexapeptide repeat domain of chicken prion protein showed low homology with those of prion protein in the mammals. In addition, the length and composition of hexapeptide repeat domain of chicken prion protein also showed significant differences in comparison of those of mammalian prion protein [30]. Thus, further study is needed to investigate the association between prion disease and hexapeptide repeat polymorphism of chicken prion protein in the future.

## Conclusion

In conclusion, the present study surveyed chicken *PRNP* polymorphisms in large samples of 4 chicken breeds, Dekalb white, Ross, Ogolgye and Korean native chickens. We found only tandem repeat deletion polymorphisms and compared the genotype and allele distribution of tandem repeat polymorphisms among 4 chicken breeds. We confirmed that chickens have significantly different genotypes and allele distributions of tandem repeat polymorphisms among 4 chicken breeds. Finally, we found significant differences in the number of *PRNP* SNPs between prion disease-susceptible species and prion disease-resistant species. To the best of our knowledge, no SNP has been identified in the chicken *PRNP* gene thus far.

## Methods

### Genetic analysis

Genomic DNA was purified from 20 mg of brain tissue sample using a Hiyield genomic DNA mini kit (Real Biotech Corporation, Taiwan). Polymerase chain reaction (PCR) was performed with chicken *PRNP* gene-specific primers: forward primer: 5′-TGGGATGATGCTTGATTTCGGT-3′ and reverse primer: 5′ ATCCCTGTCACGCTCCAGAA-3′. These primers were designed based on the chicken *PRNP* gene sequence from GenBank (Gene ID: 396452) and amplified the entire ORF of the chicken *PRNP* gene. The length of the PCR products was 978 bp. A 25 μl reaction mixture containing 2.5 μl of 10 X *Taq* DNA polymerase buffer, 1 μl of genomic DNA, 10 pmol each primer, 0.5 μl of 0.2 μM dNTP mixture, 0.2 μl of 0.04 units of *Taq* DNA polymerase and sterile deionized water to a total volume of 25 μl. The PCR conditions were as follows: denaturing at 95 °C for 2 min, followed by 34 cycles of 95 °C for 20 s, 65 °C for 30 s, and 72 °C for 1 min 30 s and 1 cycle of 72 °C for 5 min. Purified PCR products were directly sequenced using an ABI 3730 sequencer (ABI, Foster City, California, USA), and sequencing electropherograms were analyzed using Finch TV software (Geospiza Inc., Seattle, USA).

### Statistical analysis

Genotype, allele and haplotype frequencies of the chicken *PRNP* gene was compared among 4 chicken breeds by chi-square test using SAS 9.4 Software (SAS Institute Inc., Cary, NC, USA). The Hardy-Weinberg Equilibrium (HWE) test and haplotype analysis were performed using Haploview version 4.2 (Broad Institute, Cambridge, MA, USA).

### Literature search

A literature search was conducted to search for SNPs of prion protein in humans, sheep, goats, cattle, horses and chickens using PubMed. The search terms were “prion”, “SNP” combined with “human,” “sheep,” “goats,” “cattle,” “horse” or “chickens”. Moreover, we supplemented our search by screening the reference lists of the relevant studies, including original articles and reviews. References for all identified publications are indicated in Table 4.

## Supplementary information

**Additional file 1. **Sanger sequencing data of chicken *PRNP* gene in Dekalb White, Ross, Ogolgye and Korean native chicken.

## Data Availability

All data generated or analysed during this study are included in supplementary information file and available from the corresponding author on reasonable request.

## Abbreviations

BSE: Bovine spongiform encephalopathy
CWD: Chronic wasting disease
HWE: Hardy-Weinberg Equilibrium
ORF: Open reading frame
PCR: Polymerase chain reaction
*PRNP*: Prion protein gene
PrP^C^: Normal prion protein
PrP^Sc^: Misfolded prion protein
SNPs: Single nucleotide polymorphisms
TME: Transmissible mink encephalopathy
